# Extracellular vesicle encapsulated nicotinamide delivered via a trans-scleral route provides retinal ganglion cell neuroprotection

**DOI:** 10.1186/s40478-024-01777-0

**Published:** 2024-04-22

**Authors:** Myungjin Kim, Jun Yong Kim, Won-Kyu Rhim, Gloria Cimaglia, Andrew Want, James E. Morgan, Pete A. Williams, Chun Gwon Park, Dong Keun Han, Seungsoo Rho

**Affiliations:** 1 Department of Ophthalmology, CHA Bundang Medical Center, CHA University, 59 Yatap-ro, Bundang-gu, Seongnam-si, Gyeonggi-do 13496 Republic of Korea; 2 Department of Biomedical Science, CHA University, Bundang-gu, Seongnam-si, Gyeonggi-do Republic of Korea; 3 Department of Biomedical Engineering and Intelligent Precision of Healthcare Convergence, SKKU Institute for Convergence, Sungkyunkwan University (SKKU), Jangan-gu, Suwon-Si, Gyeonggi-do Republic of Korea; 4 School of Optometry and Vision Sciences, Cardiff University, Cardiff, UK; 5 School of Medicine, Cardiff University, Cardiff, UK; 6 Division of Eye and Vision, Department of Clinical Neuroscience, St. Erik Eye Hospital, Karolinska Institutet, Stockholm, Sweden

**Keywords:** Extracellular vesicle, Glaucoma, Neuroprotection, Nicotinamide, Retinal ganglion cell, Trans-scleral route

## Abstract

The progressive and irreversible degeneration of retinal ganglion cells (RGCs) and their axons is the major characteristic of glaucoma, a leading cause of irreversible blindness worldwide. Nicotinamide adenine dinucleotide (NAD) is a cofactor and metabolite of redox reaction critical for neuronal survival. Supplementation with nicotinamide (NAM), a precursor of NAD, can confer neuroprotective effects against glaucomatous damage caused by an age-related decline of NAD or mitochondrial dysfunction, reflecting the high metabolic activity of RGCs. However, oral supplementation of drug is relatively less efficient in terms of transmissibility to RGCs compared to direct delivery methods such as intraocular injection or delivery using subconjunctival depots. Neither method is ideal, given the risks of infection and subconjunctival scarring without novel techniques. By contrast, extracellular vesicles (EVs) have advantages as a drug delivery system with low immunogeneity and tissue interactions. We have evaluated the EV delivery of NAM as an RGC protective agent using a quantitative assessment of dendritic integrity using DiOlistics, which is confirmed to be a more sensitive measure of neuronal health in our mouse glaucoma model than the evaluation of somatic loss via the immunostaining method. NAM or NAM-loaded EVs showed a significant neuroprotective effect in the mouse retinal explant model. Furthermore, NAM-loaded EVs can penetrate the sclera once deployed in the subconjunctival space. These results confirm the feasibility of using subconjunctival injection of EVs to deliver NAM to intraocular targets.

**Supplementary Information:**

The online version contains supplementary material available at 10.1186/s40478-024-01777-0.

## Introduction

Retinal ganglion cell death is the signature pathophysiological event in glaucoma, reflecting the vulnerability of their axons to damage within the optic nerve head but also the impaired physiological environment within the inner retina. While the current treatment strategies are focused on reducing intraocular pressure (IOP), more than 20% of the glaucoma patients with well-controlled IOP still progress, indicating that non-IOP related factors should be addressed to improve RGC protection [50, 87]. In addition, up to 40% of treated patients go blind in at least one eye [73], making glaucoma a major health and economic burden to society. To date, the development of effective neuroprotective therapies that act in concert with the reduction of IOP has been challenging. While preclinical (animal) studies have suggested that agents such as brimonidine and memantine had potential as neuroprotectants, this was not realised in clinical trials. A significant factor in the preclinical assessment of these agents has been the reliance on using the survival of RGCs as the arbiter of success. Although, among animal models for preclinical studies, mice have advantages over rats as more genetically modifiable and more affordable in numbers, many reports have described that they are less responsive to the induction of ocular hypertension than rats. Studies in the microbead models, which are in routine use, demonstrated that mice allow less persistent and effective elevation of IOP compared to rats, which confuses the interpretation of the yield of the animal model [64]. A more sensitive method for assessing RGC health is favoured to detect subtle changes. Given the long-term nature of glaucoma, we reasoned that assessing earlier changes in RGC health status would provide a more useful index of RGC protection even in a mouse glaucoma model—since this would more easily translate to preserving vision.

Nicotinamide (NAM) treatment has been shown to provide robust protection against glaucomatous RGC damage [20, 35, 38, 92, 101, 102]. NAM supports the replenishment of cellular NAD pools, which in turn can support neuronal survival by preventing Wallerian degeneration, as NAD itself is an allosteric inhibitor of SARM1. NAD metabolism is closely linked to ATP production, the homeostasis of NAD/NADPH, and the maintenance of DNA stability [9, 93, 101]. Since mitochondrial dysfunction and ATP depletion contribute to glaucoma pathophysiology, NAM supplementation can have a positive effect on neuroprotection in glaucoma subjects [92]. Although the neuroprotective effect of NAM supplement on the retina in glaucoma subjects has been reported in many in vivo studies and randomised clinical trials [38, 103], the oral administration of NAM is considered to be a relatively less efficient method to maintain NAD levels in the retina. In previous studies, the lowest dose used was equivalent to ~ 2.7 g/day for a 60 kg human (the human dose equivalent is based on a mouse dose of 550 mg/kg/d) [102], implying that the ingestion of six tablets a day would be needed to provide effective neuroprotection against the glaucoma progression.

Direct ocular delivery is a logical approach to delivering a high dose of NAM to the retina. A variety of routes can be used, including intravitreal injection, subconjunctival (subtenon) injection, and topical (eye drop) administration. Although intravitreal injections have become routine in clinical practice, mainly for the treatment of macular degeneration, they can be painful and carry a significant risk of intraocular infection. More problematic is that injections to support retinal NAM levels would be required on a daily basis, given the short half-life of NAM. Administration of eye drops presents an attractive alternative in terms of comfort and safety. However, their efficacy is affected by patient adherence, the kinetics of transcorneal passage, dilution in the aqueous humour, and vitreous, all of which can diminish the level of NAM that reaches the inner retina. By contrast, delivery by the subconjunctival route is considered to be safer than the intravitreal injection, less limited for repeated procedures than the intracameral injection and more efficient than eye drops [1, 18, 86, 99] since if overcomes any adherence issues. In addition, it allows the delivery of depot preparations for the prolonged delivery of substances (weeks to months). Once the drug is deployed in the subconjunctival space via a small gauge needle, the sclera, the next architecture of the globe that the carriers meet, could play a role in controlling the delivery. The extracellular matrix (ECM) structure and biomechanical properties of the sclera are known to be dependent on the tissue water content, which is related to the permeability to exogenous compounds [11].

The drug delivery system (DDS) using the subconjunctival route requires biodegradable and biocompatible material-engineering technology to overcome the confounding effect of subconjunctival fibrosis, which is a hurdle for the multiple injection scheme. Low immunogeneity and low cellular toxicity are the key features of successful DDS biomaterials. Recent progress in the field of bio-inspired carriers, such as extracellular vesicles (EVs), suggests a promising solution to these issues [45, 46, 79]. EVs are cell-derived membranous structures transporting various active molecules from producer cells to recipient cells with low immune reactions to the recipients, which abilities have drawn huge attention for better therapeutic application than previous synthetic DDS platforms [8, 25, 67, 70]. Neuroprotective effect of EVs engineered by endogenous stimulation has been shown in glaucoma animal models, though issues remain with stable purification and isolation for efficacy control [79]. In that sense, we reason that the neuroprotective effect can be better stabilised by the use of exogenously engineered EVs loading proven substances such as NAM.

We report the effect of exogenously engineered EVs loading NAM delivered via a trans-scleral route on a mouse retinal explant model. We confirmed dendritic integrity as a more sensitive readout of RGC health in mice than somatic loss. Also, we demonstrate the permeability of the NAM-loaded EVs (NAM-EVs) via the trans-scleral route in vivo, suggesting that EVs provide a feasible and realistic method for the delivery of high-dose NAM to the inner retina and RGCs.

## Materials and methods

### Animal use

All experimental procedures were conducted in accordance with the UK Animals Scientific Procedures Act 1986 and the Association for Research for Vision and Ophthalmology Statement for the Use of Animals in Ophthalmic and Research. Individual study protocols complied with ethical guidelines at Cardiff (UK) and CHA (ROK) University. Animals were housed and fed in a 12 h light/12 h dark cycle with food and water available ad libitum.

Male C57BL/6 J (B6) mouse strains weighing 20–25 g were bred and used at 8–12 weeks of age for the induction of unilateral ocular hypertension (n = 13) and ex vivo retinal explant models for retinal flat mounts (n = 8, detailed below). Male New Zealand White (NZW) rabbit strains weighing 1.9–2.2 kg (n = 3) were used to evaluate the scleral penetration of DiO-labelled EV via subconjunctival injection (detailed below and in Additional file 1: Fig. 1).

### Mouse glaucoma model with microbead injections

For the induction of ocular hypertension, 17-μm diameter polyurethane (SUNPU-170, Sunjin Chemical, Gyeonggi-do, South Korea) microbeads were injected into the anterior chamber as previously described [44, 80]. Following sterilisation by ultraviolet light for 30 min, the beads were diluted in phosphate-buffered saline (PBS) at 250 mg/mL. Mice were deeply anaesthetised with an intraperitoneal injection of ketamine (37.5 mg/kg) and medetomidine hydrochloride (1.25 mg/kg) before the injection of microbeads. Microbeads were injected unilaterally into the anterior chamber (left eye) in thirteen mice, as previously described [80]. Briefly, the 1-mL syringe filled with at least 0.3 mL of microbead solution was loaded with a 30-gauge needle, which was inserted subconjunctivally 2 mm from the limbus. The tip was advanced to the limbus within the subconjunctival space before entering the anterior chamber through the iridocorneal angle. Five μL of microbead solution was then injected slowly and gently, maintaining a constant flow into the anterior chamber to minimise the outflow of beads.

### IOP measurements

IOPs were measured in both eyes preoperatively to calculate the baseline and on postoperative day 1, postoperative day 3, and on 1-week intervals up to 4 weeks after injection using a rebound tonometer (iCare TONOLAB tonometer; Tonovet, Vantaa, Finland) specifically calibrated for use with the mouse eye. All measurements were made in awake animals under gentle restraint following topical anaesthesia with 0.5% Proparacaine hydrochloride eye drops (Alcon Laboratories, Fort Worth, TX). The mice were positioned into a cloth cone with an open apex to expose the animal’s head to facilitate positioning. The IOP was measured in the right eye first, followed immediately by the left eye (treated eye). Measurements were taken for each eye within 5 s while maintaining the same body position. Six single readings were recorded, with the first and last being discarded, and the middle four measurements were averaged to provide a mean IOP estimate. The same procedure was repeated three times, and the final IOP value was defined as a mean of the three measurements. The contralateral eye was used as an untreated control. IOP recordings were always performed between 9 and 10 am to minimise the effects of the circadian rhythm on IOP.

### RGC soma counts

RGC soma loss was quantified following immunohistochemical labelling with RNA-binding protein with multiple splicing (RBPMS) of whole mount retinas. Globes were incised and fixed in 4% PFA for 2 days. Retinas were then dissected in a small dish under HBSS. Permeabilisation was carried out using 0.1% Triton-X 100 in PBS for 30 min at room temperature, followed by antigen retrieval in 300 uL of sodium citrate buffer and in a water bath at 90 °C for 30 min. Protein blocking was performed in 2.5% NHS (Normal horse serum) for 1 h. The retinas were then incubated overnight at 4 °C in a primary antibody of RBPMS Novus (1:1000) followed by two further washes in PBS before the addition of an Alexa fluor Plus tagged secondary antibody (1:1000) at room temperature for 3 h with protection from light. After 2 further washes in PBS, the retinas were stained using Hoechst (1:1000) in PBS for 30 min. Following a final rinse, the retinas were mounted with FluorSave (Merck) and coverslipped.

Images of individual RBPMS+ cells were taken using an Olympus IX71 microscope (Olympus), and four images per retina (20X magnification, 0.25 μm/pixel) were taken equidistant at 0, 3, 6, and 9 o’clock about a superior to inferior line through the optic nerve head (~ 1000 μm eccentricity). Images were cropped to 400 um^2^, and RBPMS+ cells were counted using the Cell Counter plugin for Fiji (only round nuclei were counted, thus excluding vascular endothelium); counts were averaged across the four images.

### RGC dendritic analysis

Dendritic pruning was assessed in retinal flat mounts at day 28 after the induction of ocular hypertension via DiOlistics labelling of individual cells using a custom-made gene gun as previously described [29, 92]. Bullets were prepared at a ratio of 2 mg DiI, 4 mg of DiO, and 80 mg Tungsten (1.7 μm diameter) to 30.5 cm of Tezfel tubing (Biorad), by dissolving the dyes in 400 μl of methylene chloride and applying over the tungsten to air dry. The coated tungsten was collected and transferred into the tubing and distributed along the length of the tubing by vortexing. For labelling, retinas were flat mounted onto glass slides, and all liquid was removed. A culture insert (Falcon®, pore size 3.0 μm) was inverted over the retina to act as a filter to large particle clumps, and the contents of a single bullet discharged at a gene gun pressure of 100 ~ 120 psi. Tissue was transferred to a culture dish containing Neurobasal-A media and maintained at 37 °C, 5% CO_2_ for 30 min. Retinas were then fixed in 4% PFA for 1 h, washed in PBS, and mounted with FluorSave reagent (Merck). After drying, the coverslips were sealed.

Images of individual RGC dendritic arbours were acquired on a Zeiss LSM880 confocal microscope (20X magnification, 0.45 μm/pixel, 1 μm z-thickness). A total of 182 RGCs was labelled for Sholl analysis (approximately 6 cells per retina), including 46, 47, 20, 23, 26, and 20 cells in the low IOP (n = 6, both eyes), high IOP (n = 8, both eyes), control (n = 4, 3DEV, treated), NAM (n = 4, treated), EV (n = 4, treated), and NAM-EV (n = 4, treated) groups, respectively. Whole dendritic arbours were reconstructed using Imaris (version 9.3.1, Bitplane). RGC dendrites were automatically traced using the Imaris filaments tool. Sholl analysis was performed (dendritic intersections per binned distance from the soma centre, 10 μm steps) using Imaris software to provide a quantitative index of dendritic integrity.

### EV isolation

For the generation of EV, human adipose-derived mesenchymal stem cells (ADMSCs; Lonza, Basel, Switzerland) were cultured using CellCorTM CD MSC media (CDM; Xcell Therapeutics, Seoul, Korea) with 1% antibiotic–antimycotic solution. ADMSCs were seeded 5 × 105 cells/plate on a 150 pi plate and incubated at 37 °C in a humidified environment with 5% CO_2_. The conditioning media were collected every 24 h for a total of 120 h in order to isolate EV. The collected CDM were centrifuged at 1,300 rpm for 3 min, followed by the non-exosomal large particles, such as cells, cell debris, microvesicles, and apoptotic bodies, which were eliminated using a 0.22 mm Vacuum Filter/Storage Bottle System. A 500 kDa molecular weight cut-off filter was used in tangential flow filtration (TFF; Repligen, Waltham, MA, USA) to isolate EVs. The rate of diafiltration was adjusted at 7. The Amicon Ultra-15 centrifugal Filter Unit (Merck, MA, USA) was used to concentrate the isolated EVs.

### Characterisation of EV

The MONO ZetaView® (PMX-120, Particle Metrix, Meerbusch, Germany) was used with 488 nm scatter mode to validate the quantity and size of particles. Using filtered PBS solution (HyClone laboratories, UT, USA), the EV samples were diluted to 107–108 particles/ml. For all samples, the detailed parameters for accurate analysis were tuned with sensitivity 75, shutter 100, minimum trace length 15, and cell temperature 25 °C. Transmission electron microscopy (TEM; Hitachi, H-7600, 80 kV, Japan) was used to examine the morphology of EVs. The EV solution was dried on a Formvar/copper grid with a carbon coating of 150 mesh (FCF150-CU, Electron Microscopy Sciences, USA). EVs were stained with 7% uranyl acetate or gadolinium acetate solution and dried for negative staining on a copper grid. The Formvar/copper grid was put on the grid box for TEM investigation after drying. The pH was detected by a pH meter using a microelectrode (InLab Ultra-Micro-ISM, Mettler Toledo, OH, USA) for small sample volumes.

### Western blot analysis

For the analysis of EV content, the same number of EVs (1 × 109 particles) was used. EVs were placed onto nitrocellulose (NC) membranes after 10% SDS-PAGE. The NC membrane was blocked using a TBST solution diluted in 5% skim milk. The EV protein-transferred NC membranes were incubated with CD63 (Abcam, MA, USA), TSG101, and Apo-A1 primary antibodies (Santa Cruz Biotechnology, CA, USA), followed by HRP-linked secondary antibodies (Cell Signaling Technology, MA, USA). The blot was pretreated with the enhanced chemiluminescence solution (GE Healthcare, WI, USA) and visualised with ChemiDocTM XRS + and ImageLab software (Bio-Rad, CA, USA).

### Nicotinamide loading and release profile

The isolated EVs were mixed with Nicotinamide (NAM; 500 mM), then sequentially extruded 11 times through 400 nm, 200 nm, and 100 nm polycarbonate membrane filters by using a mini extruder (Avanti Polar Lipids, AL, USA). The extruded solution was purified with the Amicon Ultra-15 centrifugal Filter Unit three times. The release profile of nicotinamide from EVs was investigated using a balanced salt solution (BSS; Alcon, TX, USA) as the release medium. One millilitre of NAM-loaded EV (NAM-EV) was packed into a 3.5 kDa dialysis membrane bag. The sealed dialysis bag was placed in 50 mL of release medium (100 rpm). 10 mL of release media was collected at regular intervals and refilled with new media. The concentration of the nicotinamide was quantified using high-performance liquid chromatography (HPLC; Vanquish VC-P20-A, Thermo Fisher Scientific, OH, USA) with an Agilent Eclipse column (5 micron, 4.6 × 150 mm C-18) at 40 °C. The mobile phase was composed of water and acetonitrile (50:50) and flowed at a flow rate of 1.0 mL min^−1^. The profile was observed at a wavelength of 210 nm.

### Mouse retina explant cultures

Mouse retinal explant cultures were prepared as detailed previously [40, 92], with a maximum of 16 retinal explants generated from additional 8 mice. Retinal flat mounts positioned ganglion cell side up in cell culture inserts (Millicell®, pore size 0.4 μm) placed into a 6-well plate. Explants were cultured at the air/medium interface and were not immersed in the culture medium. Four retinas were allocated to each group: control, EV, NAM, and NAM-EV group. With the exception of the control group, EV, NAM, and NAM-EV were dissolved in the culture medium to a concentration of 50 mM. Retinal explant cultures were maintained in humidified incubators with 5% CO^2^ at 37 °C. Half the retinal explant culture medium was changed at 1 day ex vivo (DEV). Retinas were removed from the culture after 3 DEV.

### DiO-labelled EVs inoculation

The isolated EVs were incubated with Fast DiO green fluorescent membrane dye (D3898, Invitrogen, CA, USA) at a final concentration of 2 μg/mL for 1 h at room temperature. The incubated solution was purified using a size exclusion chromatography column packed with Sepharose CL-2B (Sigma-Aldrich, MO, USA). The incubated solution was loaded on the column, and 11 fractions of 1 ml were collected. The DiO intensity was measured using a microplate reader (Molecular Devices, CA, USA) in all fractions (Excitation at 484 nm, Emission at 540 nm, Cut-off at 530 nm). The fractions 4 to 8 in the collected series were concentrated using the Amicon Ultra-15 centrifugal Filter Unit.

### Cryosection of rabbit eye

A solution containing a concentrated preparation of DiO-labelled EVs was injected subconjunctivally in the NZW rabbit’s eyes (n = 6). Animals were sacrificed either immediately, at 1 h or at 2 h following injection to investigate the depth of EV penetration into the globe at different time points. Eyes for cryo-sectioning were enucleated and washed in PBS, and blocks were immediately created using OCT compound (Sakura). Blocks were maintained at − 80 °C until use. 14 μm cryo-sections were cut (anterior to dorsal plane) using a cryotome (CM3050S, Leica) and then DAPI stained. Images were digitised using a slide scanner Axio Scan.Z1 (Carl Zeiss).

### Statistical analysis

**Fig. 1:**
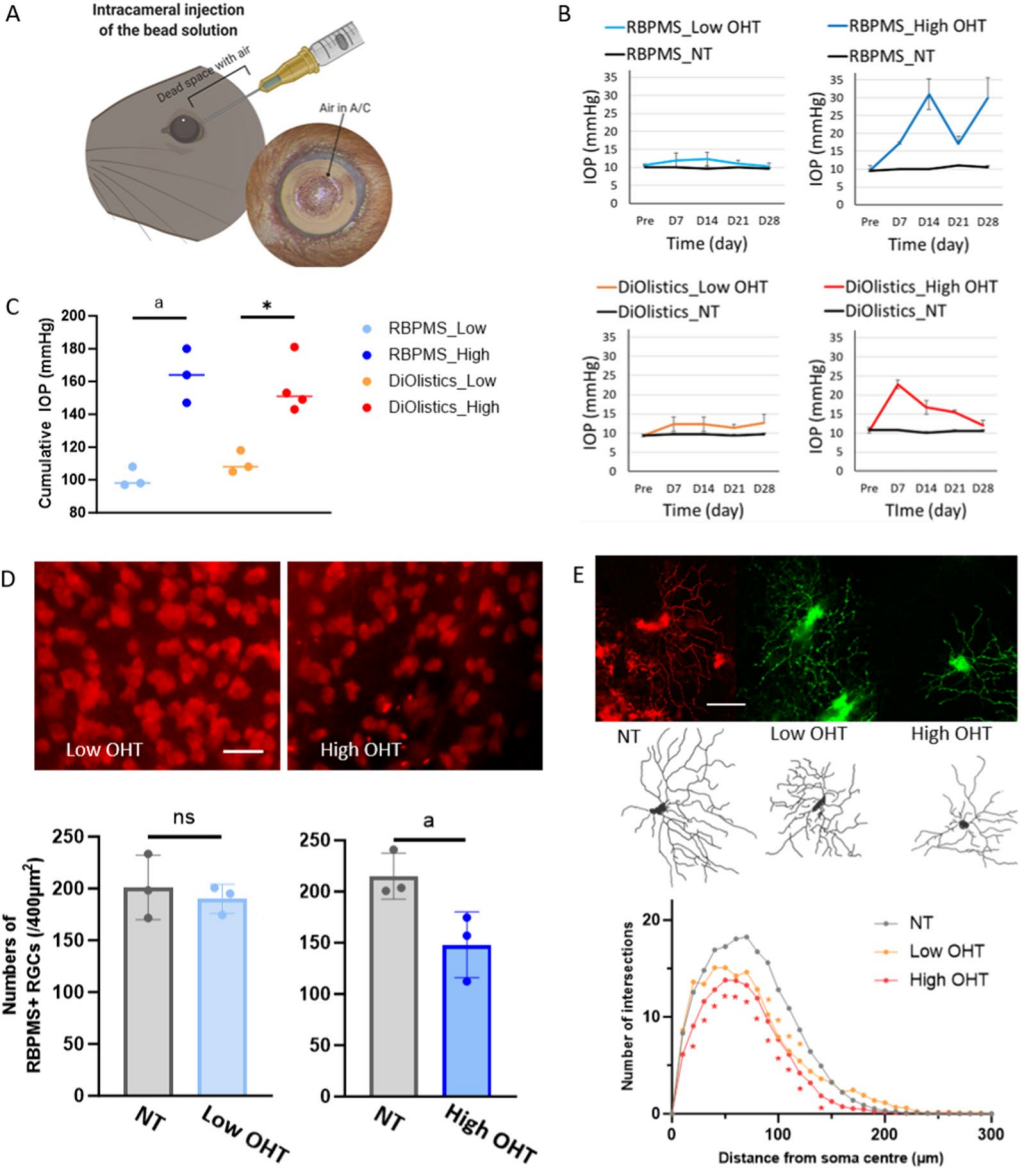
Comparison of response to IOP elevation with RGC soma counts and dendritic arbourisations in a mouse glaucoma model. **A** IOP elevation was induced using intracameral injection of polyurethane microbeads in mouse eyes (26 retinas from 13 mice). **B** IOP curves of four groups which were divided by the IOP of 17 mmHg at week 1. **C** The Cumulative IOP graph shows that the IOP elevation was statistically significant in both the RBPMS high group and the DiOlistics high group compared to the RBPMS low group and the DiOlistics low group, respectively. **D** In the RBPMS low OHT group, the count of RBPMS+ RGCs was similar to the control group. However, the count of RBPMS+ RGCs was lower, with marginal significance in the RBPMS high OHT group compared to the control group. Each value of the box plot is an average of the RGC counts in one retina. Scale bar = 20 μm. **E** Whereas the statistically significant change of dendritic arbour was apparent in both the DiOlistics low group as well as the DiOlistics high group compared to the control group. Scale bar = 50 μm. *NT* normal tension (control group), *OHT* ocular hypertension (treated group). ^a^p = 0.05, *p < 0.05, all comparison was performed between each treated group and NT

GraphPad Prism 10 was used for statistical analysis (GraphPad Software, CA, USA). Depending on the normality of data distribution (tested using Shapiro–Wilk, alpha = 0.05), Mann–Whitney U tests or independent t-tests were applied to assess group differences. P-values below 0.05 were determined statistically significant. Note that all comparisons were performed between each treated group and NT group and between each treated group and the control group (3DEV) in Figs. 1E and 3D, respectively.

## Results

### Response of retinal ganglion cell (RGC) soma counts and dendritic arbourisation in different IOP elevations

We first assessed the relationship between the level of IOP increase and changes in the two different RGC structures, RGC soma counts and dendritic arbours in a mouse glaucoma model (Fig. 1A). To find out if the level of IOP increase was a determining factor of the RGC changes, mice were divided into a low IOP increase group (6 mice) and a high IOP increase (7 mice) group depending on the level one week after microbead injection, using an IOP of 17 mmHg as the discriminate cut-off (turns out to be a subthreshold value to lead to RGC soma loss; Fig. 1B, C). A mouse model of OHT was selected to determine which methods would detect the RGC changes earlier, showing benefits in comparison among the four groups in the mouse retinal explant model described later (see in the “RGC protection with NAM-EV: ex vivo evaluation” section). Retinal flat mounts were obtained on day 28. Three retinal explants from the low OHT group and three retinal explants from the high OHT group were allocated to RBPMS to determine the level of RGC loss. For the analysis of dendritic degeneration, three retinal explants from the low OHT group and four retina explants from the high OHT group were subjected to DiOlistics labelling. Figure 1C shows the cumulative IOP for each group, confirming the sustained and moderate increase in IOP following a single injection of microbeads.

The number of RBPMS+ cells was lower in the high-pressure group compared to the control group with marginal significance (p = 0.050), whereas the number of RBPMS+ showed no significant differences in the low-pressure group compared with the control group (p = 0.500) (Fig. 1D). By contrast, Sholl analysis showed statistically significant differences in both the low-pressure group and high-pressure group compared to the control group (Fig. 1E). The observed decrease in dendritic arbourisation in response to a relatively modest increase in IOP below 17 mmHg highlights the enhanced sensitivity of evaluating dendritic pruning for detecting RGC damage caused by IOP elevation in comparison to assessing RGC soma loss.

### NAM loaded EV as a drug delivery system for neuroprotection

**Fig. 2:**
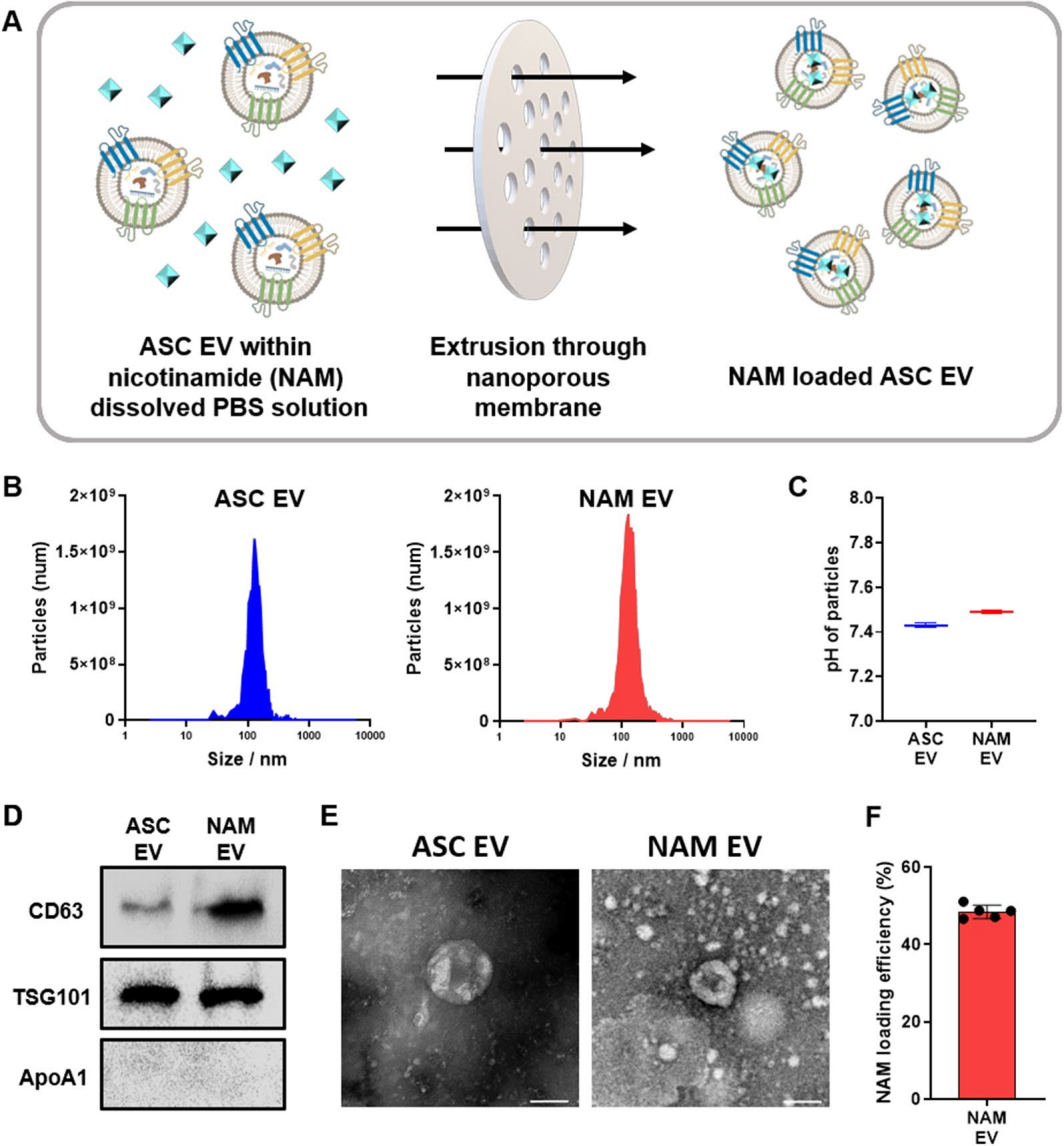
Engineering and characterisation of NAM-loaded EV. **A** Schematic illustration of the fabrication method for NAM-loaded ASC EV (NAM-EV). **B** The size distribution of both ASC EVs and NAM-EVs falls within the typical size range for EVs. **C** The pH of the ASC EV and NAM-EV were found to be neutral. **D** Confirmation by western blot of surface marker (CD63), internal marker (TSG101), and negative marker (ApoA1) of ASC EVs and NAM-EVs proved their identity as EVs. **E** The cup-shaped structural morphology of the TEM image confirmed the presence of EVs. Scale bars = 100 nm. **F** The loading efficiency of nicotinamide into EVs. *ASC* adipose-derived stem cell

The method and results of EV characterisation are shown in Fig. 2A, B. The isolated ASC-derived EV (ASC EV) and NAM-EV were quantified using the MONO ZetaView® (Fig. 2B). Mean EV size was similar for the two groups at 161 nm for ASC EVs and 155 nm for NAM-EVs. The pH values of ASC EV and NAM EV were around 7, which were suitable for eye application (Fig. 2C). Both EVs were validated with EV markers (CD63, TSG101) and were negative for the marker (ApoA1) by western blot analysis (Fig. 2D), and the typical cup-shaped morphology of EVs was identified (artefactual cup shape) with TEM in both EV types (Fig. 2E) [100, 108, 109]. These data confirm that the use of an extruder in the drug-loading process supported the reformation of particles into EV-like structures. The NAM loading efficiency was approximately 48%, with a load of 5.38 pg of NAM per particle (Fig. 2F).

### RGC protection with NAM-EV: ex vivo evaluation

**Fig. 3:**
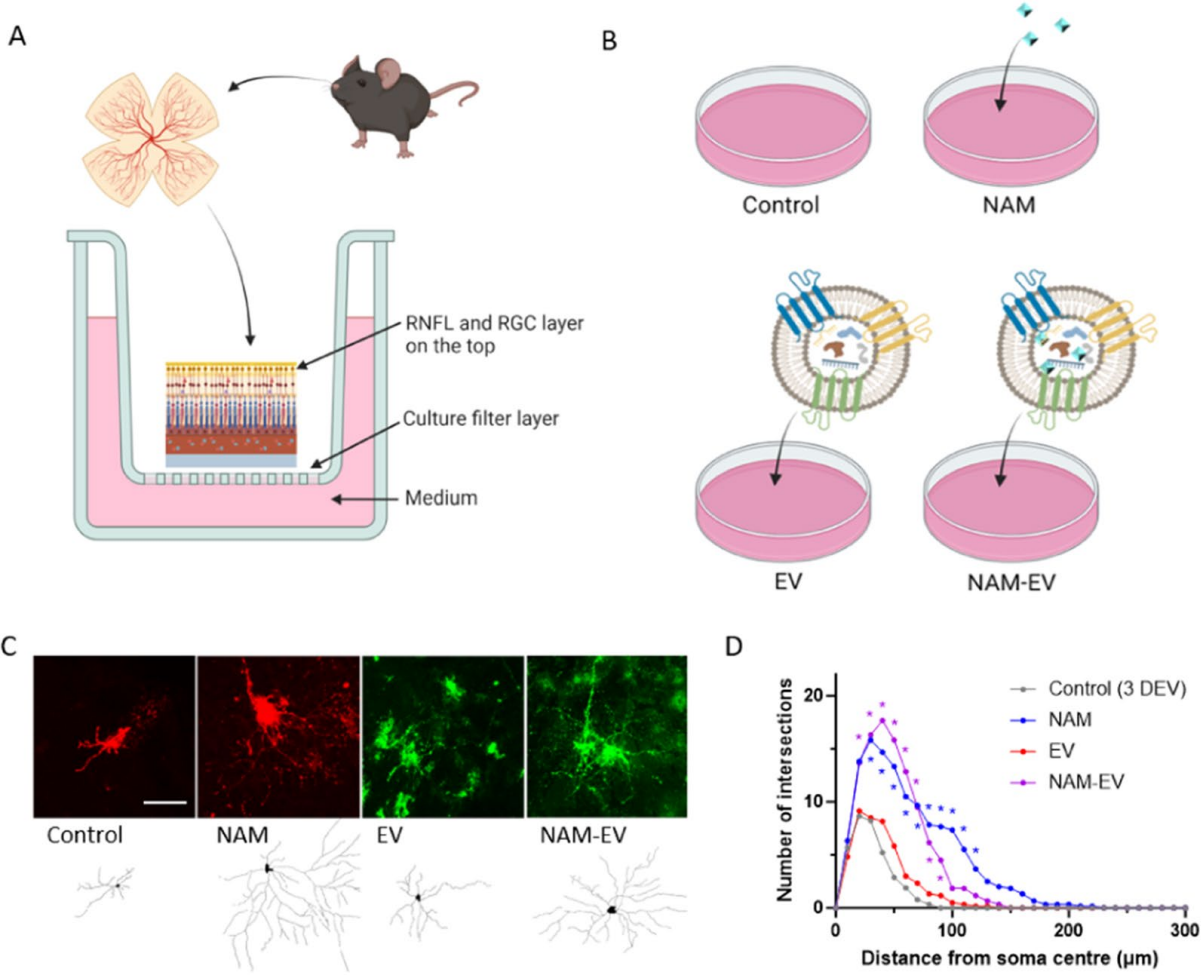
Mouse retinal explant model in four groups (control, NAM, EV, and NAM-EV). **A** Schematic illustration of mouse retinal explant and culture method. **B** Division into four groups based on the presence or absence of additives in the media: control (3DEV), NAM, EV, and NAM-EV, respectively (16 retinas from 8 mice). **C** Representative images of dendritic arbourisation in each group. Scale bar = 50 μm. **D** Sholl analysis graphs for each group show significant preservation of dendritic arbour in the NAM and NAM-EV groups compared to the control group (3DEV). *DEV* days ex vivo. *p < 0.05, All comparison was performed between each treated group and control group (3DEV)

In line with the mouse glaucoma model, media with additives (EV, NAM, and NAM-EV) were compared to control (additive-free media), all of which were passed through a cell culture insert (Millicell®, pore size 0.4 μm) to reach the whole mount retinas of mice (Fig. 3A). The control group received no additives, the EV group received EVs, the NAM group received NAM, and the NAM-EV group received a NAM-loaded EV during three days of cultures (Fig. 3B). Typical dendritic arbours for each group are shown in Fig. 3C. The area under the Sholl curve was significantly increased in the NAM and NAM-EV groups compared to the control and EV groups (Fig. 3D). Furthermore, there was no significant difference in Sholl AUCs between the control and EV groups.

### Scleral penetration of DiO-labelled EV via subconjunctival injection

**Fig. 4:**
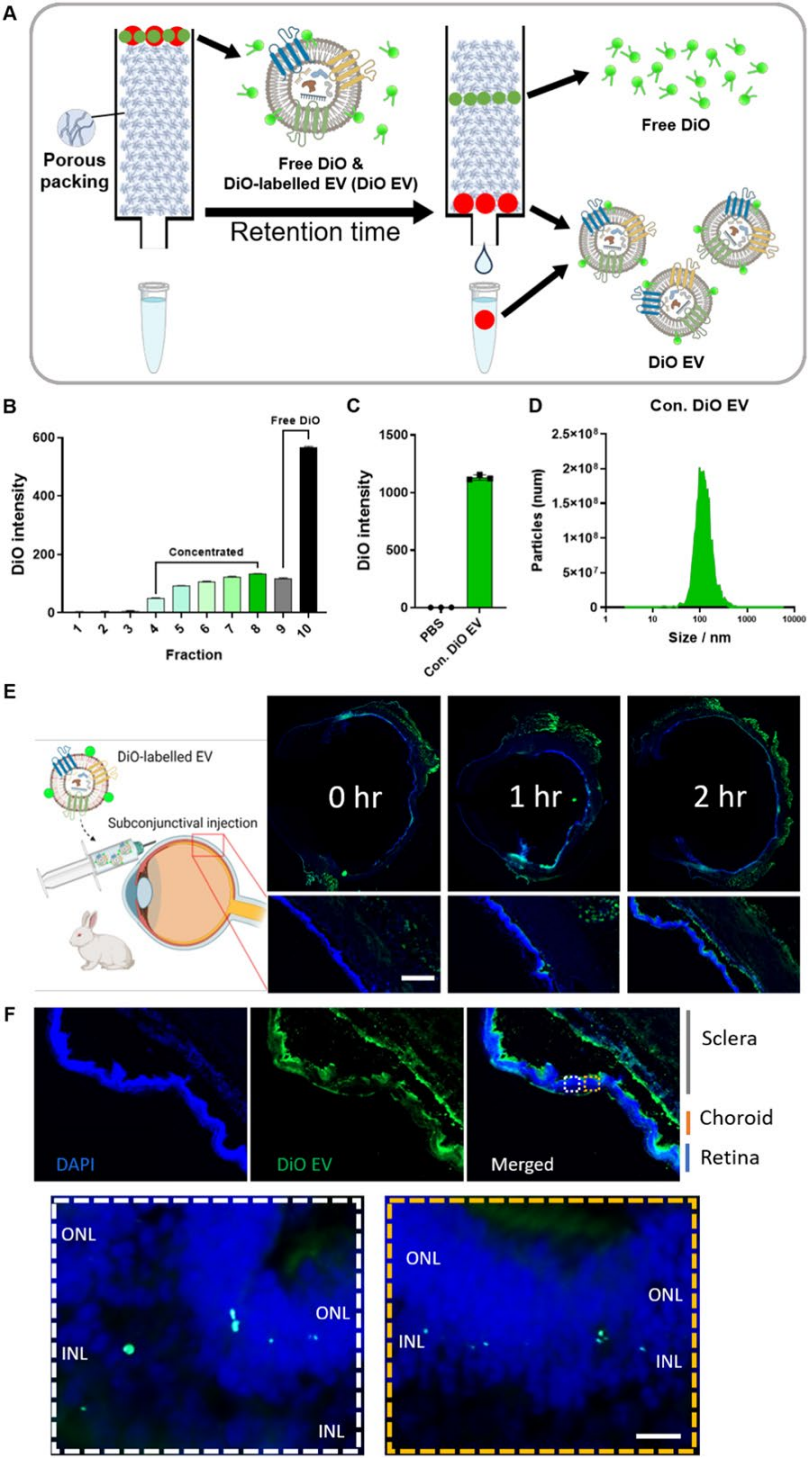
Scleral penetration of DiO-labelled EV via subconjunctival injection. **A** Schematic illustration of the purification of DiO-labelled EVs. **B** The DiO intensity of all size exclusion chromatography factions. **C** By confirming the DiO intensity of the concentrated DiO-labelled EV and PBS solutions, we proved that the concentrated DiO-labelled EV solution contained DiO-EV. **D** The size distribution of DiO-labelled EVs demonstrates they fit in the size range of EVs. **E** Detection of DiO-labelled EV penetration via sclera after subconjunctival injection in NZW rabbit. Note that the depth of EV penetration increases as time goes by (2 h > 1 h > 0 h). Scale bars = 200 μm. **F** High magnification images at 2 h after injection. The DiO-labelled EVs were detected in the retinal tissue. Scale bars = 10 μm

To explore the feasibility of utilising NAM-loaded EVs via a trans-scleral route for bigger globe size than rodents and for further research with full-scale EV production, we conducted an in vivo study in NZW rabbits to prove the scleral penetration of EV. To determine the distribution of EVs upon following subconjunctival injection, we labelled EVs with DiO, a green fluorescent, lipophilic carbocyanine dye. To eliminate the unlabelled free DiO completely, we purified the DiO-labelled EVs (DiO-EVs) using size exclusion chromatography (SEC) (Fig. 4A). Fractions eluted through the column were collected in 1 ml aliquots, and the DiO intensity of each fraction was measured. Fraction 10 exhibited a significantly higher intensity compared to other fractions, indicating the presence of free DiO within this fraction (Fig. 4B). Due to the possibility of some inclusion of free DiO in Fraction 9, it was discarded along with Fraction 10. Fractions 4–8 were deemed to contain pure DiO-labelled EVs, and a concentration process was subsequently undertaken. The concentrated DiO-labelled EVs (DiO-EVs) appeared colourless and transparent to the naked eye. When comparing the DiO intensity of the solution containing DiO-EVs to the PBS solution, the former exhibited a significantly higher intensity, indicating the presence of DiO-EVs in the solution (Fig. 4C). Furthermore, when assessing the size distribution using MONO ZetaView®, it was observed that the measured values corresponded to the typical range of EV sizes, with an average of 149 nm (Fig. 4D). No EV was detected in the retina immediately after injection, but EVs were detected in the retina 1 h after the injection (Fig. 4E, F). The labelled EVs were clearly seen in the distant part of the sclera from the injection point within a couple of hours of injection. Accordingly, this means that a single subconjunctival injection of EV could possibly deliver the cargo throughout the globe.

### NAM releasing profile from EV

**Fig. 5:**
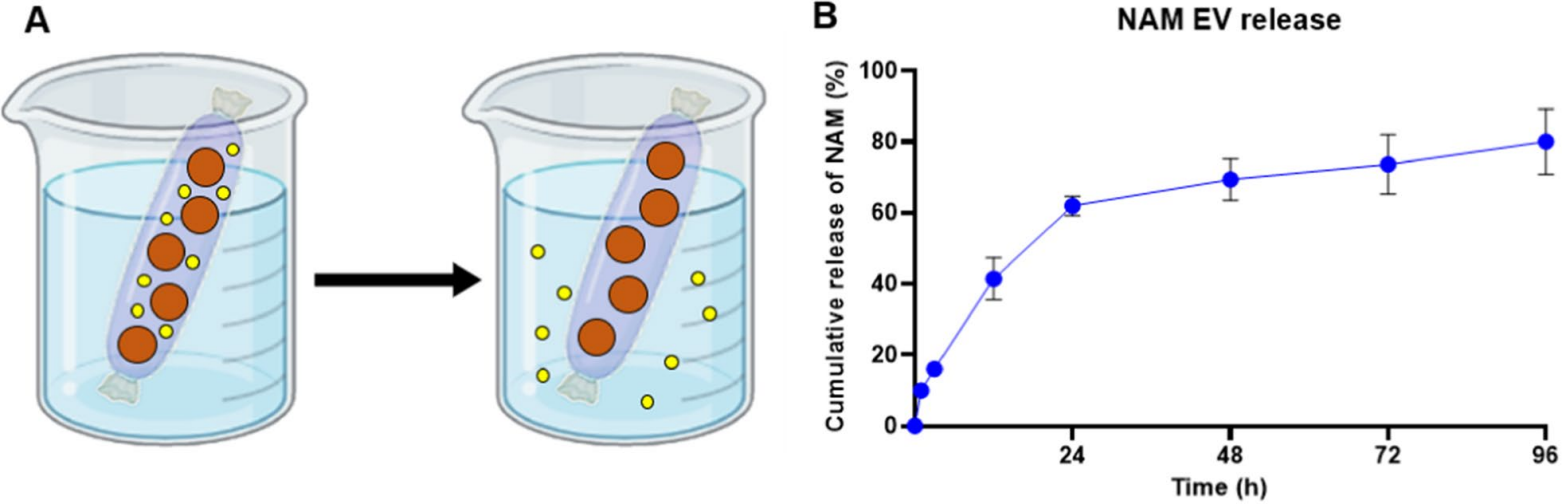
The releasing profile of NAM from EV. **A** Schematic illustration of the method for determining the nicotinamide release profile from NAM-EV. **B** The release profile of nicotinamide released from NAM-EVs showed an initial burst up to 24 h, followed by a sustained release pattern

To estimate the release kinetics of NAM from NAM-loaded EV, we employed a dialysis system [31, 77]. Using a membrane with a 3.5 kDa cut-off size, we configured the system to prevent the escape of EVs from within the membrane while allowing the release of NAM to the external environment (Fig. 5A). By converting the released drug quantities at each time point into percentages, it was observed that the data followed a first-order release kinetics model, with regression coefficients (R^2^) measuring 0.9713 (Fig. 5B). The initial burst occurred within the first 24 h, followed by a sustained release observed up to the 96-h time point. Compared to the relatively fast half-life of free NAM, NAM encapsulated inside EV can be expected to have a relatively delayed release time and a longer-lasting effect due to the low immunogenicity and longer circulation time of EV itself. Attempts are obviously needed to change the composition of the EV surface to achieve improved sustained release time in the follow-up studies[32, 58].

## Discussions

Our principal finding is that NAM-loaded EVs can reduce dendritic atrophy in an explant model of degeneration. This is confirmed using DiOlistics labelling, which is demonstrated, in our study, to be more sensitive to detecting earlier changes in a mouse glaucoma model compared to immunostaining of RGC somas. Furthermore, we demonstrated that EVs can permeate the sclera to deliver NAM for enhancing efficiency, allowing further consideration of EV engineering in this field.

Dendritic pruning is a characteristic event in degenerating neurons in a variety of diseases [57]. A previous study using human postmortem retinal tissue with severe glaucomatous damage demonstrated that RGCs showed a reduction in dendritic complexity and field size, which precedes changes in cell body size compared to normal tissue [72]. This implies that dendritic pruning could be an earlier change of neurodegeneration than somatic loss of RGCs and their axons [15]. We compared the susceptibility of the RGC counts on RBPMS+ cells and the dendritic changes with staining by DiOlistics to the IOP elevation insult in a mouse glaucoma model. Although the RGC count was reduced with marginal significance in those eyes with high IOP, this did not occur in eyes with more moderate IOP elevation. In the demonstration, we still observed dendritic atrophy, which confirms the utility of using dendritic degeneration as a sensitive and robust readout of RGC degeneration.

However, RGC counts based on retrograde labelling using Fluorogold or DTMR better reflect the degree of axonopathy than immune-labelled RGC counts. A study has shown that in DBA/2 J mice, the cell body persisted following severe depletion of retrograde axonal transport of fluorogold [13]. Similarly, in a laser-induced mouse model with a transient and rapidly elevated IOP, RGC bodies stained by selective markers outnumber the cells labelled retrogradely from the colliculus. The losing speeds of labelled RGC numbers in both methods were also different [83, 97]. However, iatrogenic axon damage and progressing degeneration by complete labelling are inevitable in the antegrade or retrograde labelling of RGC, which can be criticised. With the DiOlistic method, we confirmed that the application of NAM-loaded EVs in the medium has a neuroprotective effect in a retinal explant model comparable to NAM-applied medium being free from these issues.

Then, we explored the feasibility of the EVs as drug carriers and whether NAM can deliver into the scleral shell to reach the retinal tissue via the transscleral route. The pearl of our study is that we have found how fast and far the EVs can travel with a single subconjunctival injection in vivo for the first time. In our study, EVs can be delivered throughout the globe, permeating into the sclera within a couple of hours. In terms of sustainability, the half-life of NAM in the plasma is very short (1.5 h with 1g dose). Still, in theory, chemical drugs can be protected from the in vivo environment through the EV formulation [32, 91]. There is a report that EV lasts for four weeks with intravitreal injection thus NAM-EV may have a lasting effect compared to free NAM [58]. Once EVs reach the retinal tissue via intravitreal injection, they can penetrate the inner and outer retina in a scattered fashion and reached to choroidal tissue [75]. Similarly, we found that the EVs used in our study were scattered in the retinal tissue.

Although drugs to the retina can be delivered in other ways, such as intravitreal injection, systemic administrations and topical applications, they are partially limited to particular reasons. Intravitreal injections are an efficient method for delivering drugs into the retina and are currently a widely utilised modality in many retinal diseases [74]. As reported in the literature, serious adverse events of intravitreal injection include endophthalmitis, retinal detachment, lens injury, and inflammation, all of which are relatively rare [28, 33, 51, 71]. However, the patients’ satisfaction and quality of life may be more affected by ocular discomfort caused by the injection itself, as some patients are reluctant to undergo painful procedures. Pain associated with the procedure affects patients’ compliance with treatment. A report demonstrated that scoring pain with the visual analogue scale (VAS) ranged from 0 to 84 (a mean of 17.4 ± 17.1) in patients receiving bevacizumab (Avastin; Genentech Inc, South San Francisco, CA) injections [62]. Although the ANCHOR and MARINA studies have led to improved clinical care [12, 81], the need for regular injections can be burdensome from the patients’ compliance perspective. A less invasive method of delivery would have potential pros in terms of reduced cost, patient compliance, and side effects [88]. Systemic delivery can be hindered by the blood retinal and blood aqueous barriers. High drug concentration is usually required to overcome these barriers to diffusion, with the potential risk of systemic side effects [5]. Some drugs or carriers can be degraded in the liver, which also creates a high demand for drug intake in the first place [78, 89]. Topical administration is safer and easier than other methods except for compliance issues. Even though the topical route has obvious advantages, delivery to the retina is difficult for several reasons, such as lacrimation, relatively low corneal permeability, counter-directional intraocular convection, and most weightily, the long distance for diffusion [23].

Contrary to these potential obstacles of other routes, the transscleral or subconjunctival route has clear advantages [6, 7, 59, 68]. Patient acceptance and attitude toward the subconjunctival route is relatively high; 61.6–74.2% were acceptable with subconjunctival injection, whereas 57.2% were acceptable with intracameral injection[17, 19], which might be related to the concerns about the higher risks of infection and pain in intravitreal or intracameral injection. Due to the large and accessible surface area and a high degree of hydration with hypocellularity, the sclera is conducive to water-soluble substances, and proteolytic enzymes are less likely to degrade drugs [88]. Since the sclera is similar to the corneal stroma anatomically, the higher permeability of the sclera than the cornea has motivated researchers to study transscleral drug delivery that needs to be delivered to the back of the eye [24, 68, 76].

Forming around more than three-quarters of the outer tunic of the human eyeball, the sclera is a remarkably resilient and structurally complex connective tissue that performs multiple functions critical to vision [11]. Primarily, the sclera provides a stable and firm structure for the retina, avoiding off-axial light emission from outside the globe. The scleral matrix consists of collagen and elastin fibres, and the posterior scleral fibres are less tightly packed than the anterior sclera, which is beneficial for the transscleral delivery into the posterior retina. The arrangement of these fibres and the presence of the negatively charged proteoglycan in the scleral matrix is thought to influence drug diffusion [5]. Previous studies have reported that the sclera is more permeable to hydrophilic molecules than lipophilic ones [2, 24]. Other drug-related factors such as molecular weight, shape, conformability, and molecular radius may influence diffusion across the scleral tissue [88]. Scleral permeability decreased with increasing molecular weight and molecular radius. Molecular radius was a better predictor of scleral permeability than molecular weight [6]. The effect of molecular weight and radius has been relatively well studied, but fewer studies have reported that of surface charge, molecular shape, and conformability. The term, molecular conformability or deformability represents the structural transformation that occurs when certain molecules enter a distinguished physicochemical milieu [96], which is not fully understood.

Since clearance and tissue distribution profiles of the DDS carriers are mainly governed by the vehicle characteristics, synthetic DDS platforms such as liposomes, micelles, nanoparticles, and biodegradable polymers were studied in this field [3, 14, 61, 85, 95, 106]. Of these, the most extensively studied vehicle is the liposome, which was released on the market with the approval of the Food and Drug Administration (FDA) in 1995 [10]. Liposomes are composed of a lipid bilayer, allowing it not only to accommodate hydrophobic substances but also to capture hydrophilic ones in the internal aqueous phase [84]. Notwithstanding the advantages of liposomal formulations, there are many hurdles for the delivery of drugs. The rapid clearance of liposomes via the reticuloendothelial system (RES), and their accumulation in liver and spleen hinders reaching the target tissues [56, 84]. Moreover, the excessive accumulation of liposomes in macrophages can influence the phagocytic activity, resulting in immune suppression [22, 54, 90] and also can activate an acute hypersensitivity reaction: complement activation-related pseudoallergy (CARPA) leading to the release of inflammatory cytokines [84]. A latanoprost-loaded implant with a biodegradable platform made of biodegradable poly-lactic-co-glycolic acid (PLGA) was used for intermediate-term follow-ups for the assessment of the safety of rabbit eyes. Microscopic observations of the implant sites at 3 and 9-month animals found fibrous encapsulation and infiltrates of inflammatory cells, including macrophages and lymphocytes around and within the subconjunctival space containing implants [39]. These findings can hamper the feasibility of the biodegradable materials which ought to be deployed in the subconjunctival space for repeated therapeutic regimens. One of the ways to detour the limits of synthetic biodegradable platforms is to utilise natural carrier systems, for example, EVs.

The potential features of EVs to reduce toxicity and foreign body reaction as a DDS platform are among the important reasons why they have been more favoured recently. EVs are to be more minimally reactive to the immune system due to their biological origin compared to synthetic materials. EVs are relatively safe, as they are not mutagenic and non-replicative, following no neoplastic changes, which are confirmed by many in vivo studies and clinical trials [16, 30, 34, 36, 42, 52, 82, 111].

In order to utilise EVs as drug envelopes, a precondition is to enhance the loading efficiency. Two different methods for EV loading, exogenous and endogenous engineering techniques, can be used [54, 94]. EVs can be exogenously engineered for drug carriers by simple incubation [112], electroporation [4], sonication [47], freeze-thawing and extrusion [37], with variable degrees of success. However, all of these loading techniques can cause the aggregation of EVs accompanying physicochemical and morphological changes [48].

We have found that NAM can be successfully loaded in the exogenously engineered EVs to reach the effective doses to offer the neuroprotective effect against the neurodegeneration event. As the EV repeatedly passes through the membrane to encapsulate free drug, all types of surface proteins are rearranged randomly, which can be evidenced by the different intensities of CD63, a representative tetraspanin [27, 104]. Exogenous EV engineering is free from causing the bias influenced by the donor cells, allowing the fair comparison of the neuroprotective effect between the NAM-loaded EV group and the NAM only group. However, the production of the NAM-loaded EVs at a clinical practice level is on a different note. Upscaling the production of EVs to therapeutic levels depends on cell culture conditions, isolation and purification methods. So far, the efforts for EV production have focused on increasing scale at small to medium size with larger numbers of flasks, sizing up of cell factories or bioreactors, all of which produce up to 8–10 L of conditioned medium [26, 49, 60, 66, 98, 110]. Still, these efforts are in their infancy, and it is important to decide early on approaches capable of producing sufficient quantities of EVs for the intended clinical development programmes and subsequent in-market supply. In isolation, tangential flow fractionation (TFF) combined with ultracentrifugation (UC) methods is the most commonly used protocol, whereas bead-elute chromatography is scalable, ultracentrifugation is difficult to scale up and problematic to scale out [26, 34, 65, 66, 98]. Despite a crude method that just precipitates EVs rather than purifies them, the precipitation may still be a viable method for EV production on a large scale. As safety and quality control are the most critical issues for large-scale purification and isolation for preclinical, clinical or industrial-level production, viable features of the methods can be considered more weighty than absolute purity [25].

Supplementation of NAD-promoting EVs in various routes can deliver a neuroprotective effect on the target tissue. Since the levels of extracellular nicotinamide phosphoribosyltransferase (eNAMPT) decline with ageing in mice and humans, increasing the level of eNAMPT can counteract ageing and extend the health span. Supplementation of eNAMPT-containing EVs improved physical activity and extended lifespan in mice [105]. Local delivery of NMN preconditioned EVs promoted infarct healing through the improvement of angiogenesis by miR-210-3p via targeting the EFNA3 in a rodent animal model for myocardial infarction [105]. To the best of our knowledge, our study is the first report that the delivery of NAM using EVs to promote RGC survival in a retinal explant model is feasible. However, the release profile of NAM from our engineered EVs showed relatively rapid clearance of NAM, which needs further research related to sustained drug delivery techniques. The release profile of NAM can be controlled by exosome engineering through hybridisation with other biomaterials such as liposomes. Due to limitations for modifying the lipid composition of exosomes, the characteristics of engineered exosomes can be controlled by adjusting the lipid composition of liposomes during the hybridisation process for exosomes and liposomes [55, 63]. Differences in lipid composition determine the fluidity and rigidity of liposomes, which also affects the release profile of the encapsulated drug [21, 69]. The incorporation of cholesterol or sphingomyelin into liposomes and aromatisation within the lipid acyl chain provide rigidity to the bilayer of liposomes and reduce the permeability of encapsulated drugs to the exterior region [41, 53]. Additionally, the use of external stimuli-responsive lipids for controlling drug release from liposomes has been extensively studied. The incorporation of thermo- or pH-sensitive lipids controlled the release of loaded drugs in a specific environment [43, 107]. Phasing in hybridisation of EV engineering could be helpful and feasible for the larger scale EV production for the in vivo models, where our following experiments are headed.

## Conclusion

By all accounts, EVs hold great potential as a novel DDS platform, especially for delivery of NAM into the RGCs via transscleral route. Our data suggests that the ability of EVs to cross physical barriers like sclera and EVs reaching the outer retina can deliver NAM into the RGCs in the inner retina, as shown by the hindering dendritic pruning. These can offer us highly encouraging proof-of-concept for preclinical models. Obstacles that need to be hurdled include upscaling of the EV production and isolation. The delicate control of the drug-releasing profile from the EVs should also be investigated more in future research.

### Supplementary Information

**Additional file 1: Fig. S1**. Number of animals used.

## Data Availability

All data generated or analysed during this study are included in this published article.

## Abbreviations

ASC: Adipose-Derived Stem Cell
ATP: Adenosine Triphosphate
DDS: Drug Delivery System
EFNA3: Ephrin-A3
eNAMPT: Extracellular Nicotinamide Phosphoribosyltransferase
EV: Extracellular Vesicle
IOP: Intraocular Pressure
miR: MicroRNA
NAD: Nicotinamide Adenine Dinucleotide
NAD+: Nicotinamide Adenine Dinucleotide (oxidised form)
NADH: Nicotinamide Adenine Dinucleotide (reduced form)
NADP+: Nicotinamide Adenine Dinucleotide Phosphate (oxidised form)
NADPH: Nicotinamide Adenine Dinucleotide Phosphate (reduced form)
NAM: Nicotinamide
NMN: Nicotinamide Mononucleotide
RGC: Retinal Ganglion Cell
TEM: Transmission Electron Microscopy
TSG101: Tumor Susceptibility Gene 101
ApoA1: Apolipoprotein A1
