# Supplementary figures and images for: Extracellular vesicle encapsulated nicotinamide delivered via a trans-scleral route provides retinal ganglion cell neuroprotection
Source: Acta Neuropathol Commun. 2024 Apr 22;12:65. doi: 10.1186/s40478-024-01777-0 (PMC11036688; doi:10.1186/s40478-024-01777-0)

**Supplementary Figure 1. Number of animals used**


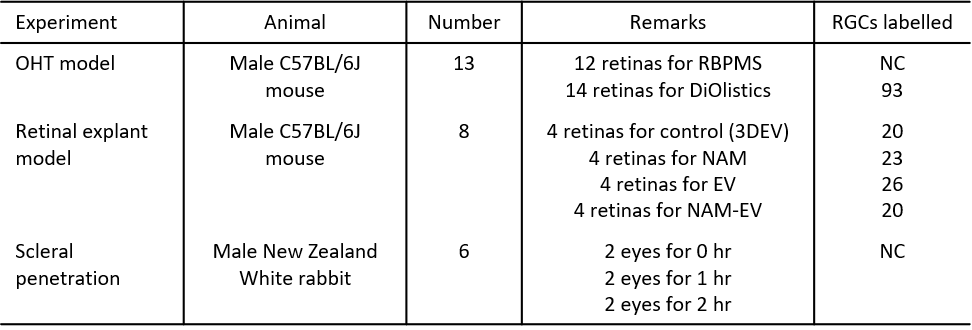

Supplement: Supplementary file 1 — Additional file 1: Fig. S1. Number of animals used. [file 40478_2024_1777_MOESM1_ESM.docx]
